# Supplementary material for: Regulation of X. laevis M18BP1 centromeric localization and CENP-A assembly
Source: bioRxiv. 2025 Jul 15:2025.07.15.664882. Preprint. [Version 1] doi: 10.1101/2025.07.15.664882 (PMC12338635; doi:10.1101/2025.07.15.664882)
Supplement: Supplement 1 [file NIHPP2025.07.15.664882v1-supplement-1.pdf]

Figure EV1

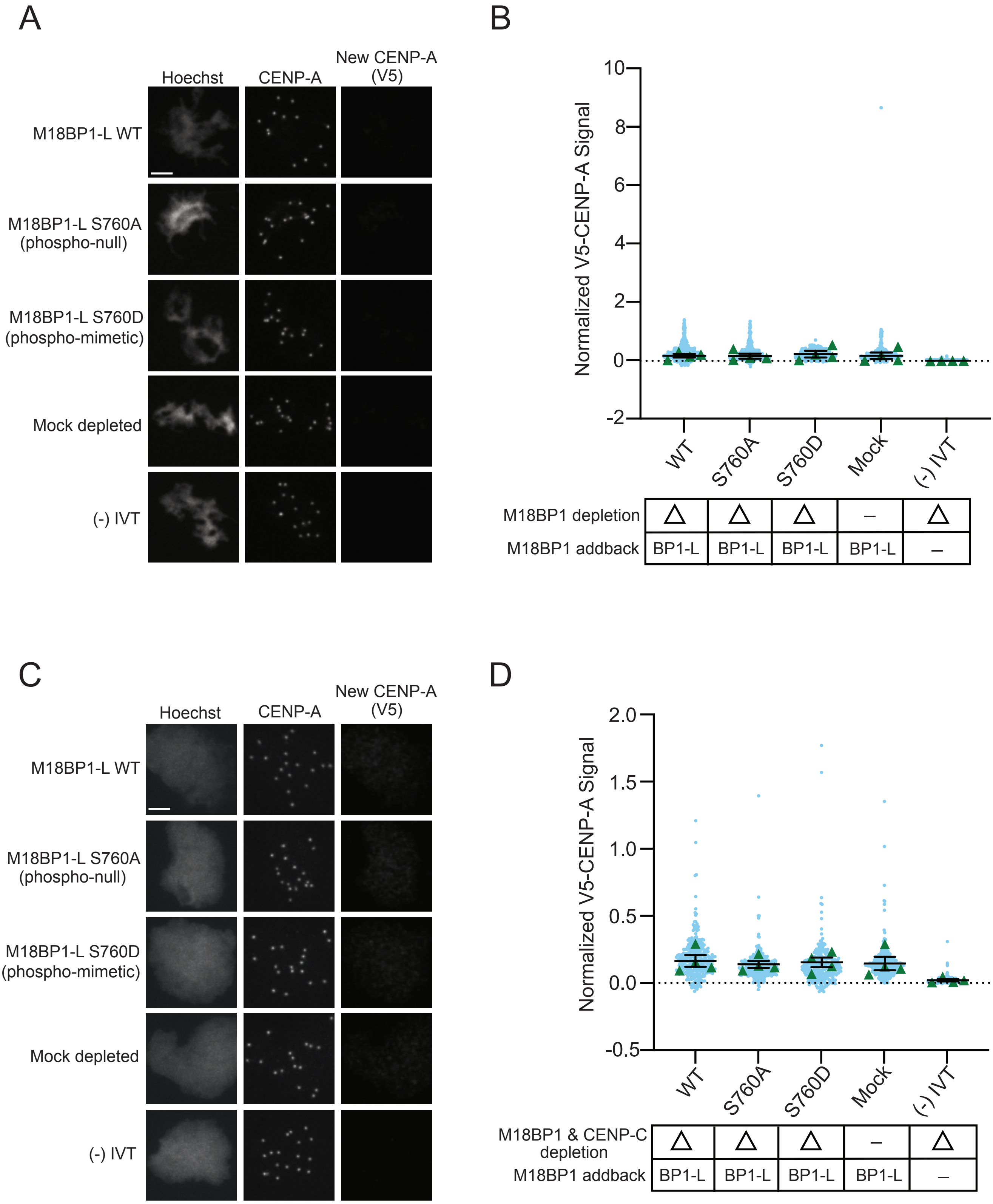

A
Hoechst
CENP-A
New CENP-A (V5)
M18BP1-L WT
M18BP1-L S760A (phospho-null)
M18BP1-L S760D (phospho-mimetic)
Mock depleted
(-) IVT
B
Normalized V5-CENP-A Signal
10
8
6
4
2
0
-2
WT
S760A
S760D
Mock
(-) IVT
M18BP1 depletion
M18BP1 addback
BP1-L
C
Hoechst
CENP-A
New CENP-A (V5)
M18BP1-L WT
M18BP1-L S760A (phospho-null)
M18BP1-L S760D (phospho-mimetic)
Mock depleted
(-) IVT
D
Normalized V5-CENP-A Signal
2.0
1.5
1.0
0.5
0.0
-0.5
WT
S760A
S760D
Mock
(-) IVT
M18BP1 & CENP-C depletion
M18BP1 addback
BP1-L

bioRxiv preprint doi: https://doi.org/10.1101/2025.07.15.664882; this version posted July 15, 2025. The copyright holder for this preprint (which was not certified by peer review) is the author/funder, who has granted bioRxiv a license to display the preprint in perpetuity. It is made available under aCC-BY 4.0 International license.

Figure EV1

A. Representative immunofluorescence images of new V5-CENP-A assembly in metaphase extract immunodepleted of endogenous M18BP1 then supplemented with full-length WT or mutant FLAG-M18BP1-L or a mock depletion or (-) IVT control (indicated on left). Labeling for DNA (Hoechst), total CENP-A, and new CENP-A (V5) is indicated above the image. Scale bar is 5μm.

B. Quantification of new V5-CENP-A with controls (indicated below) in metaphase egg extract immunodepleted of endogenous M18BP1. M18BP1 depletion and addback condition is indicated in the bottom table. The signal is normalized to the WT FLAG-M18BP1-L addback condition. Error bars represent SEM of four independent replicates (n = 4) with green triangles displaying the mean of each replicate and blue circles representing each individual centromere.

C. Representative immunofluorescence images of new V5-CENP-A assembly in metaphase extract immunodepleted of endogenous CENP-C and M18BP1 then supplemented with full-length WT or mutant FLAG-M18BP1-L or a mock depletion or (-) IVT control (indicated on left). Labeling for DNA (Hoechst), total CENP-A, and new CENP-A (V5) is indicated above the image. Scale bar is 5μm.

D. Quantification of new V5-CENP-A with controls (indicated below) in metaphase egg extract immunodepleted of endogenous CENP-C and M18BP1. CENP-C and M18BP1 depletion and addback condition is indicated in the bottom table. The signal is normalized to the WT FLAG-M18BP1-L addback condition. Error bars represent SEM of four independent replicates (n = 4) with green triangles displaying the mean of each replicate and blue circles representing each individual centromere.
